# Supplementary material for: Hierarchical Nanoflowers of MgFe2O4, Bentonite and B-,P- Co-Doped Graphene Oxide as Adsorbent and Photocatalyst: Optimization of Parameters by Box–Behnken Methodology
Source: Int J Mol Sci. 2022 Aug 26;23(17):9678. doi: 10.3390/ijms23179678 (PMC9455985; doi:10.3390/ijms23179678)
Supplement: Supplementary file 1 [file ijms-23-09678-s001.zip › ijms-1867449-supplementary.pdf]

# Hierarchical Nanoflowers of $MgFe_2O_4$, Bentonite and B-,P- Co-Doped Graphene Oxide as Adsorbent and Photocatalyst: Optimization of Parameters by Box–Behnken Methodology

**Manpreet Kaur Ubhi [1], Manpreet Kaur [1,*], Dhanwinder Singh [2], Mohammed Javed [3], Aderbal C. Oliveira [4], Vijayendra Kumar Garg [4] and Virender K. Sharma [5,*]**

[1] Department of Chemistry, Punjab Agricultural University, Ludhiana 141001, Punjab, India
[2] Department of Soil Science, Punjab Agricultural University, Ludhiana 141001, Punjab, India
[3] Department of Mathematics, Statistic and Physics, Punjab Agricultural University, Ludhiana 141001, Punjab, India
[4] Institute of Physics, University of Brasilia, Brasilia 70000-000, Brazil
[5] Program for Environment and Sustainability, Department of Environmental and Occupational Health, School of Public Health, Texas A&M University (TAMU), College Station, TX 77843-1266, USA
* Correspondence: manpreetchem@pau.edu (M.K.); vsharma@tamu.edu (V.K.S.)

**S1 FT-IR analysis**

FT-IR spectrum of MGF-B (Figure 4a) exhibited broad bands at 3400 and 1633 $cm^{-1}$ corresponding to the O-H stretching and bending vibrational modes of water molecules. The two prominent bands at 435 and 565 $cm^{-1}$ were indicative of the intrinsic stretching vibration of M-Omoiety in the $O_h$ and $T_d$ sites of the spinel ferrite nanostructure, respectively. Distinct broad band at 1048 $cm^{-1}$ was observed because of Si-Al-O framework vibrations of bentonite clay.

The major bands in FT-IR spectrum of BPGO (Figure 3a) are at 3424, 2904, 1618 and 1099 $cm^{-1}$. These bands are ascribed to O-H, CH/$CH_2$, C=C and C-OH stretches, respectively. The band at 1375 $cm^{-1}$ ascribed to the symmetric B-C/B-O/C-O vibrations. The O-B-O bending was observed for the vibration band in the range 784-622 $cm^{-1}$. Also the bands at 1440, 1216, 932, 548-463 $cm^{-1}$ corresponded to C-P stretching, P-O-P symmetric stretching, P-O stretching/P-OH bending/P-O-P asymmetric stretching and P-O-C bending vibrations, respectively. It confirmed the boron and phosphorous co-doping in GO network.

The FT-IR spectrum of HNFs exhibited all the absorption bands of pristine MGF-B and BPGO along with some red shifting in the position of bands due to change in the coordination environment (Figure 3a). Also weakening of peak intensities was depictedsignifying the interaction of BPGO with MGF-B. Similar, shift in the band positions was also described by Narayan et al. [48] and Zhou et al. [49].

**S2 Thermal analysis**

A TGA method was utilized to explore the thermal stability of the synthesized nanocomposites and BPGO and the results are shown in Fig 3b. For MGF-B, a constant weight loss of 3.81% was detected till 891°C. This may be due to evaporation of surface and lattice adsorbed water molecules. In case of BPGO, TGA curve exhibit a single mass loss step, when the weight loss reaches approximately 14% (at 440°C), the mass of the samples

declines rapidly upto 533°C, indicating the beginning of the decomposition. The fabricated nanostructure exhibit a higher thermal stability than BPGO and a slow weight loss was observed with increase in temperature from 25°C to 900°C. This may be due to presence of lesser quantity of thermal-labile oxygenated functional groups than BPGO. Further, the MGF-B can dissipate heat more fast than that of BPGO, and subsequently improves the thermal stability of fabricated nanostructure.

However, compared to the weight loss percentage of MGF-B and BPGO, nanofabricated composite exhibited higher weight loss.This is explained by the fact that hybrid materials undergo breakdown at a higher temperature and include connected oxygen functionality.The order of thermal stability was: MGF-B > HNFs > BPGO. Differential thermogravimetric curves in which the temperature at maximum weight loss rate ($T_{max}$) is obtained. The main mass loss occurred from 560-596°C, 56°C and 464-502°C for BPGO, MGF-B and HNFs, respectively.

**S3 Evaluation of best nanoadsorbent**

The synthesized materials were studied for the removal of Pb(II) ions and it observed that HNFs displayed the highest adsorption efficiency (96%) followed by BPGO (90%) and MGF-B (87%) (Figure S2a). The advantage of using HNFs as adsorbent is that co-doped GO layers provided matrix to MGF-B and mass production of HNFs was easier than MGF-B. In the HNFs, the accumulation of MGF-B and co-doped GO layers restacking was attenuated on interactions of BPGO layers with MGF-B, which further reduced the requirednanoadsorbentdosage for heavy metal ions adsorptive removal than pristine compounds. On increasing in the MGF-B content in the HNFs had a positive influence

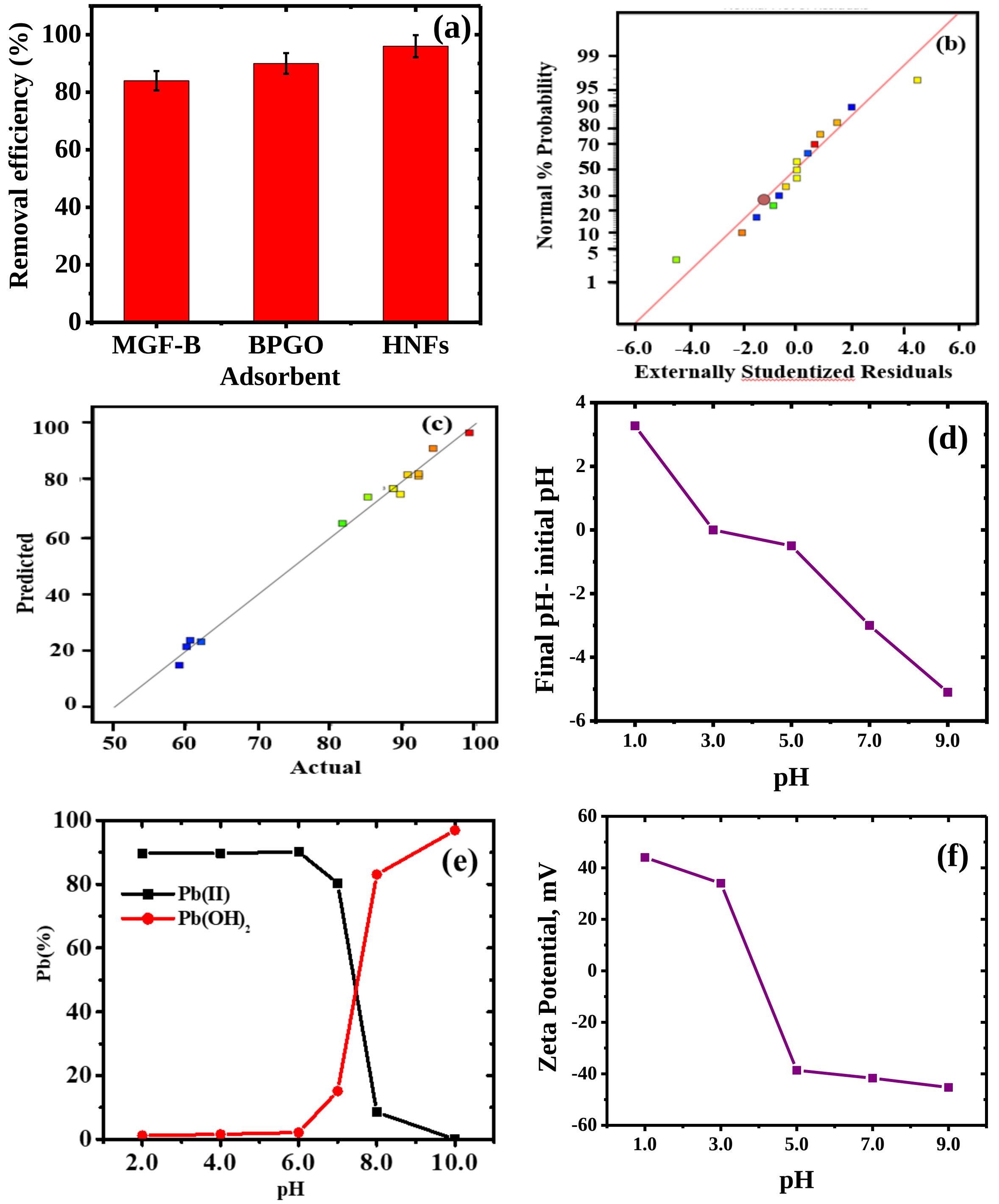


**Figure S2. Removal efficiency of MGF-B, BPGO and HNFs for Pb (II) ions (5 mg $L^{-1}$,100 mL) solution using 0.2 g $L^{-1}$ of adsorbent at 25 °C, 120 rpm, for 2 hours, Plots of (b) residuals normal probability (c) predicted vs. actual relationship, (d)zero point charge, (e) speciation curve of Pb(II) ions and (f) Zeta potential of HNFs**

on adsorption capacity upto a w:w ratio of 1:1 due to increased interactions of BPGO nanosheets which may physically hindered partial active sites for the adsorbing solutes. On further increasing MGF-B content in HNFs, negative influence on the adsorption capacity was

observed. This may be due to high magnetic character as high $M_s$value results in greater agglomerates of MGF-B which decreases the surface area of HNFs and ultimately decreases its adsorption capacity.

**S4 Kinetic modelling**

To evaluate rate of adsorption on Pb(II) ions and the dynamics involved in adsorption process, the pseudo-first order, pseudo-second order, Elovich and intra-particle diffusion kinetic models in linear and non-linear forms were applied. Table S1 shows the calculated parameters and error functions of the kinetic models to differentiate among linear and non-linear forms.

The pseudo-first order kinetic model was considerably distracted from linearity and high statistical metrics predicted its non-suitability (Figure S3). Using HNFs, the value of $q_e$ for Pb(II) ions in linear and non-linear method was 7.44 and 22.88 mg $g^{-1}$, respectively. As a result, the $q_e$ values obtained using the two methods varied greatly.

Pseudo-second order kinetic model (Figure 7b), where the adsorption rate is proportional to the twice of the active sites available on the HNFs surface, depicted that experimental data tremendously fit the equation with high $R^2$ value and there was good correlation between experimental and theoretical $q_e$ values. Using HNFs, the $q_e$ value for Pb(II) ions in linear and non-linear method was 23.58 and 23.52 mg $g^{-1}$, respectively.

In Elovich kinetic model (Figure S3b), higher α value than β showedgreater adsorption than desorption rate. The value of α calculated from linear and non-linear kinetic models was same *i.e.* $5.5 \times 10^8$ mg $g^{-1}$.min. The value of β also follows the similar trend *i.e.* for linear and non-linear kinetic models of Pb(II) ions, its value was same i.e. 1.0 g $mg^{-1}$, respectively. Elovich and pseudo-second order kinetic models deliberated adsorption as a solitary process, it isn’t relevant to distinguish the contribution of diffusion to the general adsorption rate.

Intra-particle diffusion model was utilized for this and expressed as a function of the $t^{1/2}$(Figure S3c).The intercept didn't passes through the origin, indicating adsorption occurred by both film and pore diffusion. The value of A calculated from linear and non-linear kinetic models for Pb(II) was same *i.e.* 20.57 mg $g^{-1}$.min, respectively.

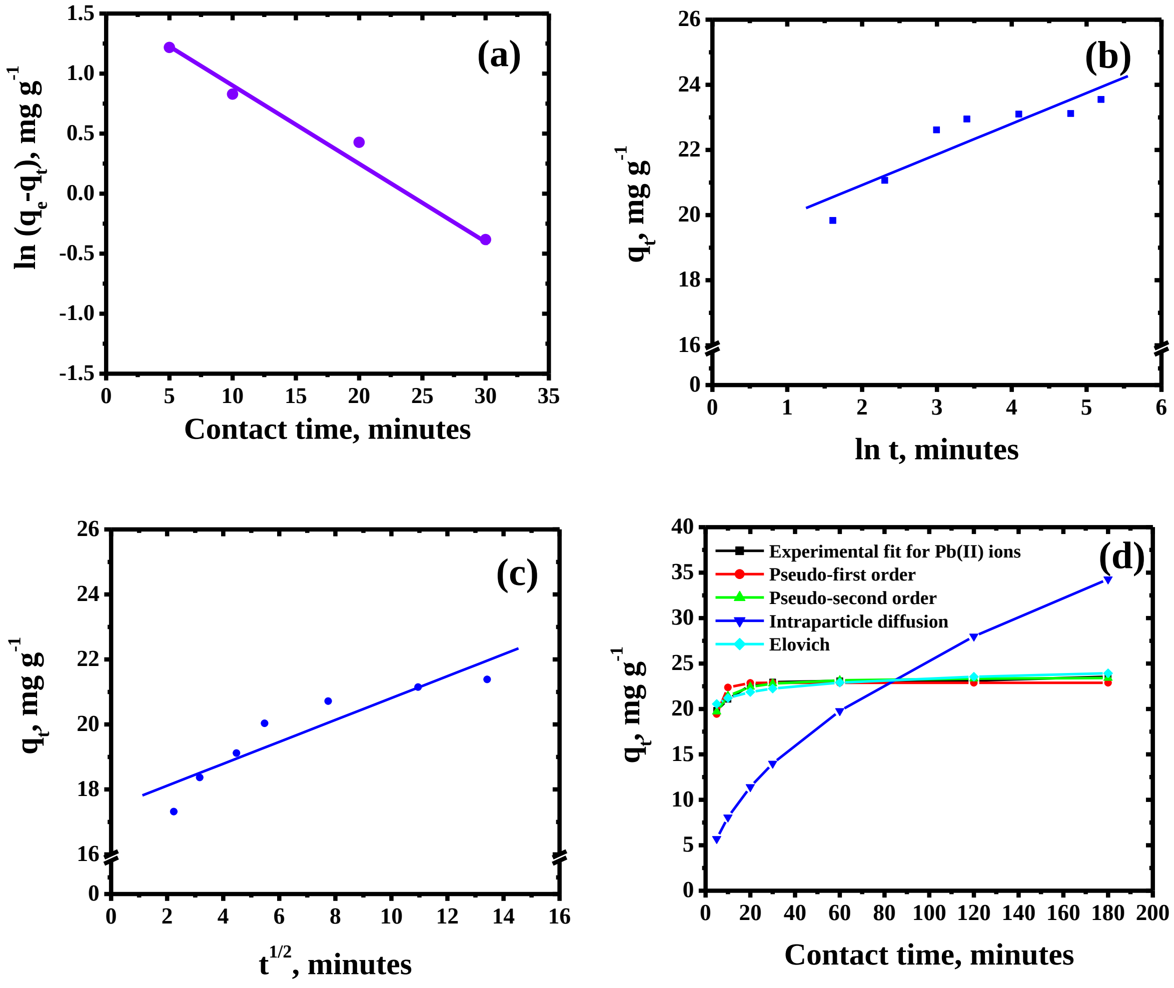


**Figure S3 Linear kinetic modelling of (a) pseudo-first order, (b) Elovich models, (c) intraparticle diffusion, (d) non-linear kinetic modelling using HNFs (0.4 g $L^{-1}$) at pH-6.0 for Pb(II) ions (100 mL each) at 25 °C, 120 rpm**

**Table S1: Kinetic parameters for Pb(II) adsorption using HNFs as adsorbent**

| Kinetic models | Parameters | Linear | Non-Linear |
|---|---|---|---|
| Pseudo-first Order | $q_e$ [a] | 7.44 | 22.88 |
| | $k_1$ [b] | 0.06 | 0.37 |
| | SSE [c] | 0.03 | 0.01 |
| | $\chi^2$ [d] | 0.05 | 0.04 |
| | RMSE [e] | 0.09 | 0.07 |
| Pseudo-second order | $q_e$ [a] | 23.58 | 23.52 |
| | $k_2$ [f] | 0.03 | 0.05 |
| | SSE [c] | 0.001 | 0.002 |
| | $\chi^2$ [d] | 0.11 | 0.21 |
| | RMSE [e] | 0.04 | 0.05 |
| Elovich | α [h] | $5.5 \times 10^8$ | $5.5 \times 10^8$ |
| | β [i] | 1.0 | 1.0 |
| | SSE [c] | 1.98 | 1.99 |
| | $\chi^2$ [d] | 0.08 | 0.09 |
| | RMSE [e] | 0.51 | 0.53 |
| Intra-particle diffusion | $k_{id}$ [g] | 0.25 | 2.55 |
| | A | 20.57 | 20.57 |
| | SSE [c] | 4.15 | 2237.07 |
| | $\chi^2$ [d] | 0.19 | 48.65 |
| | RMSE [e] | 0.77 | 17.87 |

[a-] uptake capacity at equilibrium time (mg $g^{-1}$), [b-] PFO rate constant ($min^{-1}$), [c-] sum of square of errors, [d-] Chi-square, [e-] residual root square error, [f-] PSO rate constant(g $mg^{-1}$.min), [g-] intra-particle rate constant (L $g^{-1}$.mg.$min^{1/2}$), [h-] initial adsorption rate (mg $g^{-1}$.min), [i-] desorption constant (g $mg^{-1}$)

## S5 Adsorption isotherm modelling

Different two-parameter Langmuir, Freundlich, Dubinin-Radushkevich (DR) and Temkin adsorption isotherm models in linear and non-linear forms were utilized to determine the interaction mechanism between metal ions and adsorbent. The parameters of various adsorption isotherm models are explained in Table S2.

Using linear Langmuir isotherm plot (Figure 7c), HNFs displayed $q_{max}$ and bvalue of 654.23 mg $g^{-1}$ and 0.13 L $mg^{-1}$, respectively. From non-linear form (Figure S4d), the $q_{max}$ and b values for Pb(II) ions was 745.36 mg $g^{-1}$ and 0.12 L $mg^{-1}$, respectively. The Separation factor (R) values in the range 0-1 indicated the favorable nature of the adsorption process.

The value of $K_f$ in Freundlich adsorption isotherm model was 52.29 [(mg/g)/(mg/L)$^n$)] and the same for non-linear form was 81.36 [(mg/g)/(mg/L)$^n$)] (Figure S4a). The n value was in the range of 1.58-1.83 indicated favorablenature of adsorption process (Fan et al 2008).

The value of E in DR isotherm was < 8000 J $mol^{-1}$, indicating that physisorption takes place (Figure S4b). The $q_{max}$ valuefor Pb(II) ions was 234.72 mg $g^{-1}$ and the same for non-linear form was 355.38 mg $g^{-1}$.

The A value in Temkin adsorption isotherm was 2.69 L $g^{-1}$ and the same for non-linear plot was 2.68 L $g^{-1}$, indicating that Pb(II) ions possessed more binding energy with HNFs surface (Figure S4c).

**Table S2: Adsorption isotherm parameters**

| | Parameters | Linear | Non-Linear |
|---|---|---|---|
| **Langmuir isotherm** | $q_{max}$ [a] | 654.23 | 745.36 |
| | $b$ [b] | 0.13 | 0.12 |
| | $R$ [c] | 0.59 | 0.61 |
| | SSE [d] | 0.003 | 0.008 |
| | $\chi^2$ [e] | 0.03 | 0.06 |
| | RMSE [f] | 0.008 | 0.01 |
| **Freundlich isotherm** | $K_F$ [g] | 52.29 | 81.36 |
| | $n$ [h] | 1.83 | 1.58 |
| | SSE [d] | 0.23 | 0.55 |
| | $\chi^2$ [e] | 0.14 | 0.34 |
| | RMSE [f] | 0.14 | 0.22 |
| **Dubinin-Radushkevich isotherm** | $q_{max}$ [a] | 234.72 | 355.38 |
| | $\beta*10^{-7}$ [i] | 2.24 | 9.19 |
| | $E$ [j] | 2112.88 | 1043.13 |
| | SSE [d] | 6.84 | 11.21 |
| | $\chi^2$ [e] | 2.36 | 9.26 |
| | RMSE [f] | 2.64 | 6.88 |
| **Temkin isotherm** | $A$ [k] | 2.69 | 2.68 |
| | $B$ [l] | 96.23 | 96.39 |
| | $b$ [m] | 25.74 | 25.70 |
| | SSE [d] | 11451.71 | 11453.23 |
| | $\chi^2$ [e] | 22.81 | 23.51 |
| | RMSE [f] | 32.21 | 33.02 |

[a]- Maximum adsorption capacity (mg $g^{-1}$), [b]- affinity constant (L $mg^{-1}$), [c]- separation factor (dimensionless), [d]- sum of square of errors, [e]- Chi-square, [f]- residual root square error, [g]- Adsorption capacity [(mg/g)/(mg/l)$^n$)], [h]- Freundlich intensity constant (dimensionless), [i]- Coefficient associated with energy ($mol^2$ $kJ^{-2}$), [j]- Polanyi potential ($kJ^2$ $mol^{-2}$), [k]- Temkin binding constant (L $g^{-1}$), [l]- Heat of adsorption constant (kJ $mol^{-1}$), [m]- Temkin isotherm constant (kJ $mol^{-1}$)

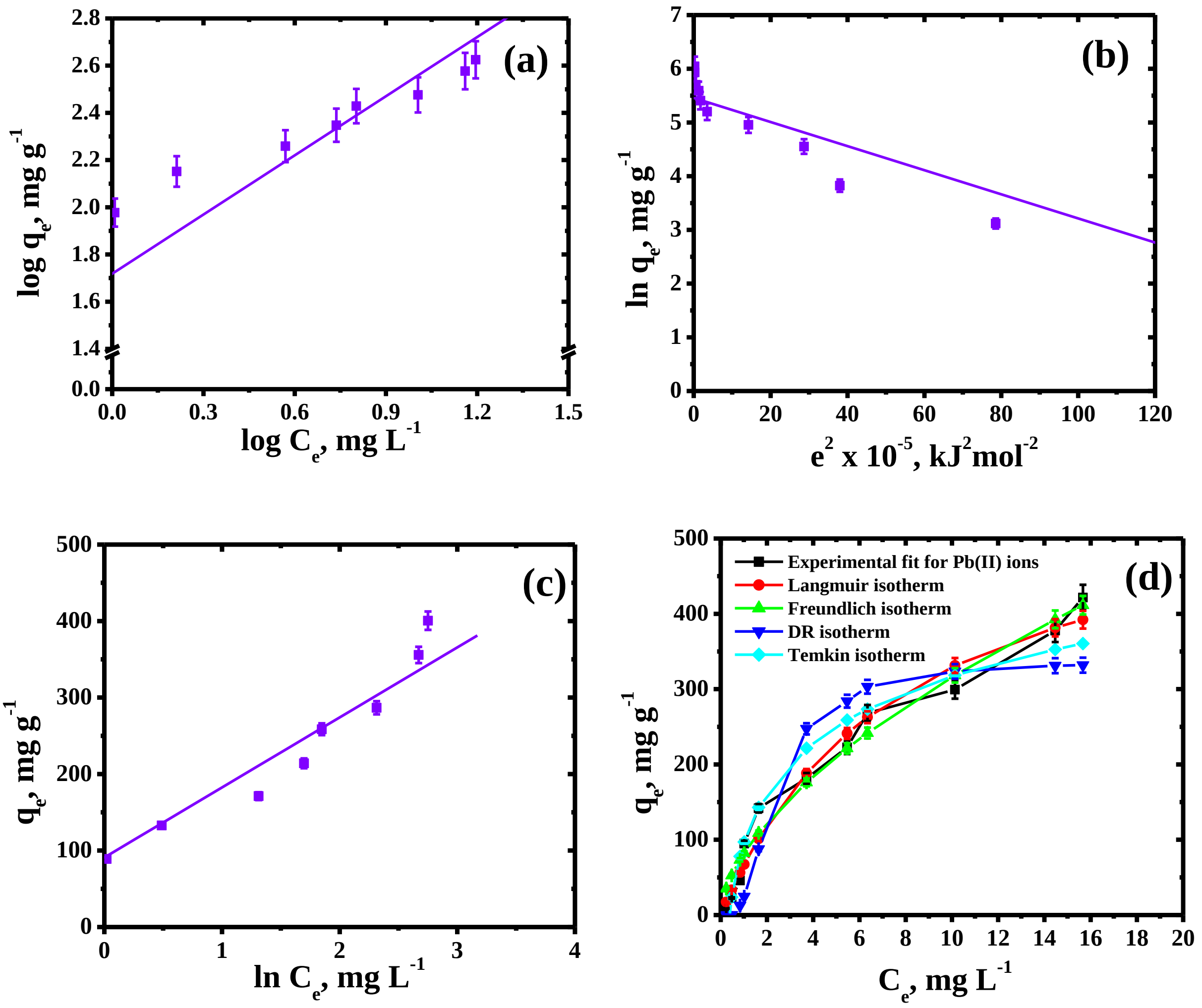


**Figure S4 Linear adsorption modelling of (a) Freundlich, (b) DR, (c) Temkin isotherm models, (d) Pb(II) non-linear isotherm modelling using HNFs at 25 °C, 120 rpm**

## S6. Characterization techniques

X-ray photoelectron spectra (XPS) of synthesized materials was obtained on a X-ray photoelectron spectroscope model PHI 5000 Versa Probe III. The Mössbauer analysis of the synthesized magnetic materials was carried out at 25°C using a Wissel coupled to $^{57}Co$ in Rh matrix in transmission and constant acceleration mode. Brunauer-Emmett-Teller (BET) analysis was performed on Quantachrome Nova-1000 surface analyzer. Thermal analysis were carried out using TGA using SII 6300 EXSTAR. Fourier transformation infrared (FT-IR) spectra were

recorded using a Perkin Elmer spectrum RX-I FT-IR spectrophotometer in the range of 4000 – 400 $cm^{-1}$. X-ray diffraction (p-XRD) patterns were recorded on a PanalyticalX'pert Pro equipped with $CuK_\alpha$ radiation ($\lambda$= 1.54 $A^o$). The topographic features of synthesized materials were analysed by Transmission Electron Microscopy (TEM) using a Hitachi Hi-7650 with a 100kV accelerating voltage. Scanning Electron Microscopic (SEM) images were collected on a Hitachi S-3400 under ambient conditions and their Electron Dispersive Spectra (SEM-EDS) were recorded using point and shoot method at 15 kV on thermo Noran System SIX. Hysteresis loops for MGF-B and HNFs were recorded at 298 K using vibrating sample magnetometery (Model PAR-155) by applying an external magnetic field of ± 10kOe. Fluorescence spectra were obtained using Agilent Cary Eclipse Fluorescence spectrophotometer.Tauc plot of $(\alpha h\nu)^2$*vs.* energy of photon (hν) was used to determine the band gap of materials.

After adsorption, the samples were centrifuged (Remi) and the content of residual metal ion was calculated on Thermo Electron Corporation's Emission Spectrometer, iCAP 6300 using online hydride generation kit. The Nano-ZS Zeta Sizer Nano Series instrument was used to determine the value of ξ- potentials. For the determination of point zero charge ($pH_{ZPC}$), 10 mg of synthesized compounds was mixed with 50 mL of 0.01 M NaCl aqueous solution having pH ranging from 2.0 to 10.0 and shaken for overnight at 25°C at 120 rpm. The final pH of the samples was recorded and $pH_{ZPC}$was determined by plotting a graph between initial pH and ΔpH ($pH_{final}$ – $pH_{initial}$). The remaining concentration of malathion after photodegradation was studied by a UV-Vis spectrophotometer (UV-1800 Shimadzu UV-visible) at the wavelengths of 250 nm. The degradation of organic pollutants and evolution of their hydroxylated intermediates and end products were also monitored by gas chromatography-mass spectrometry (Thermo Scientific TSQ 8000 Gas Chromatograph - Mass Spectrometer). The values of limit of detection (LOD) and quantification (LOQ) for Pb(II) and malathoion solutions were evaluated as:

$$LOD = 3\ (s/S)$$

$$LOQ = 10\ (s/S)$$

Here, S = slope of the calibration curve and s = standard deviation of replicate measurements having lowest concentration in the calibration curve. For the present work, the respective LOD and LOQ values for Pb(II) solution were determined to be 0.01 and 0.003 ppm, while that of malathion solution were $1.7 \times 10^{-3}$ and $5.1 \times 10^{-3}$ ppm.

**S7. Adsorption experiments**

The impact of coexisting cations was studied in the quaternary system of Pb(II), Cd(II), Zn(II) and Ni(II) with 100 mL of 0.1 mM solution of metal ions and 0.4 g $L^{-1}$ of HNFs at pH 6.0, 25°C for 2 hours. Whereas the influence of anions *viz.* $Cl^-$, $NO_3^{2-}$, $SO_4^{2-}$, $CO_3^{2-}$ and $PO_4^{2-}$ ions having concentration ranging from 0.1 to 10.0 mM prepared using their sodium salts on the adsorption of 0.1 mmol$L^{-1}$, 100 mL of each Pb(II) ions was investigated using 0.4 g$L^{-1}$ of adsorbent at 25°C for 2 hours.

Reusability studies were carried out using 0.2 g of HNFs in 100 ml of 5 mg$L^{-1}$Pb(II) solutions. The prepared solutions were agitated for 2 hours at 120 rpm at 25°C and centrifugates were analysed to determine the concentration of Pb(II) ions. After that, the spent adsorbent was collected, dispersed, and then regenerated with 0.1 M HCl. The treated adsorbent was used for next adsorption experiment.

**S8. Photodegradation experiments**

For the quenching experiments, 1.0 mL (10 mM) each of methanol, ascorbic acid, sodium azide, and disodium ethylene diaminetetraacetate were added to each flask containing 50 mL of malathion (2 mg L-1, pH-9.0). After 120 minutes of visible light photocatalysis, the solution was subjected to spectrophotometer examination.

Six successive cycles of photodegradation were used to test the HNFs' ability to be recycled. In this experiment, 100 mL of a malathion solution with optimum pH and 0.1 g of photocatalyst added to it. To achieve adsorption-desorption equilibrium, the solutions were

agitated for two hours at 130 rpm before being exposed to visible light irradiation. The samples were centrifuged and the centrifugates examined for any changes in concentration after two hours. The photocatalyst was repeatedly washed with deionized water after each cycle was finished. For the subsequent cycle of photocatalysis, the photocatalyst was recovered by centrifugation and dried at 60 °C in an oven.

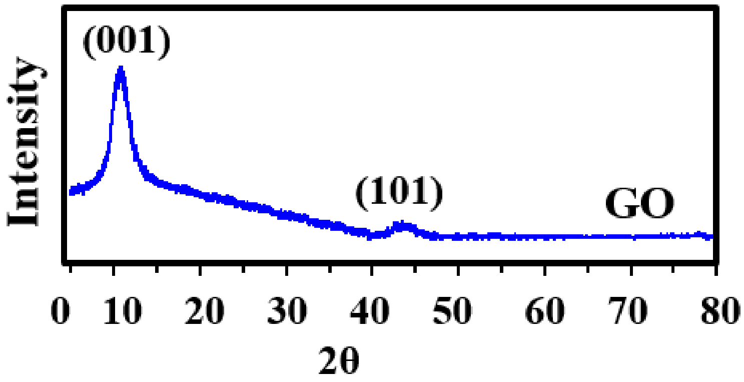


**Figure S1** XRD pattern of graphene oxide (GO)

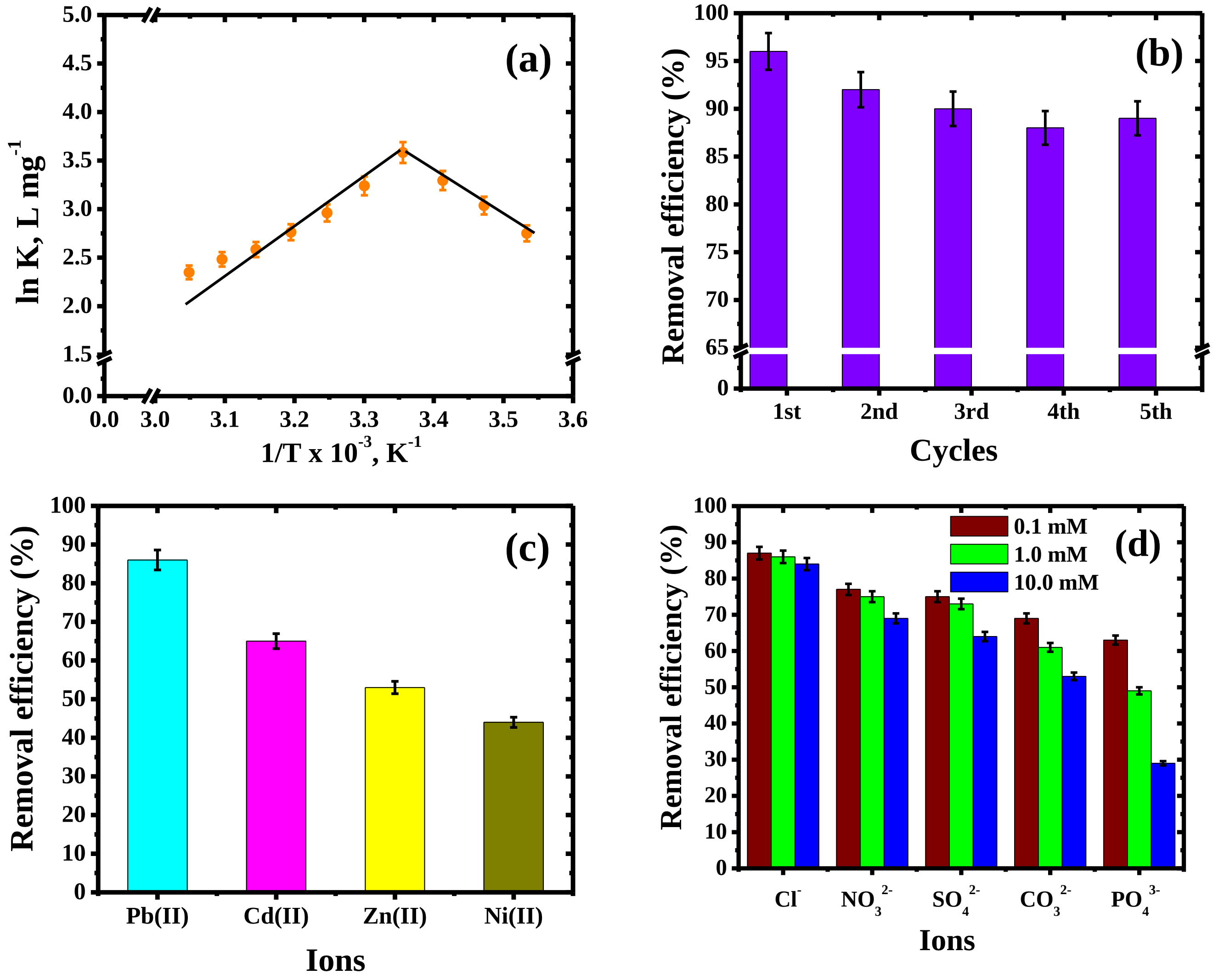


**Figure S5 (a) Vant' Hoff plot, (b) Desorption efficiency of HNFs for Pb(II) ions (Experimental conditions: optimum pH, pollutant concentration-2.0 mgL$^{-1}$, adsorbent dose-0.4 gL$^{-1}$), (c) Effect of multi ions on the adsorption of Pb (II) solution (5 mg L$^{-1}$, 100 mL) using HNFs at optimum pH, 25 °C, 120 rpm for 120 minutes of contact time and (d) Impact of coexisting anions on Pb(II) ion adsorption using HNFs at 25 °C, 120 rpm**

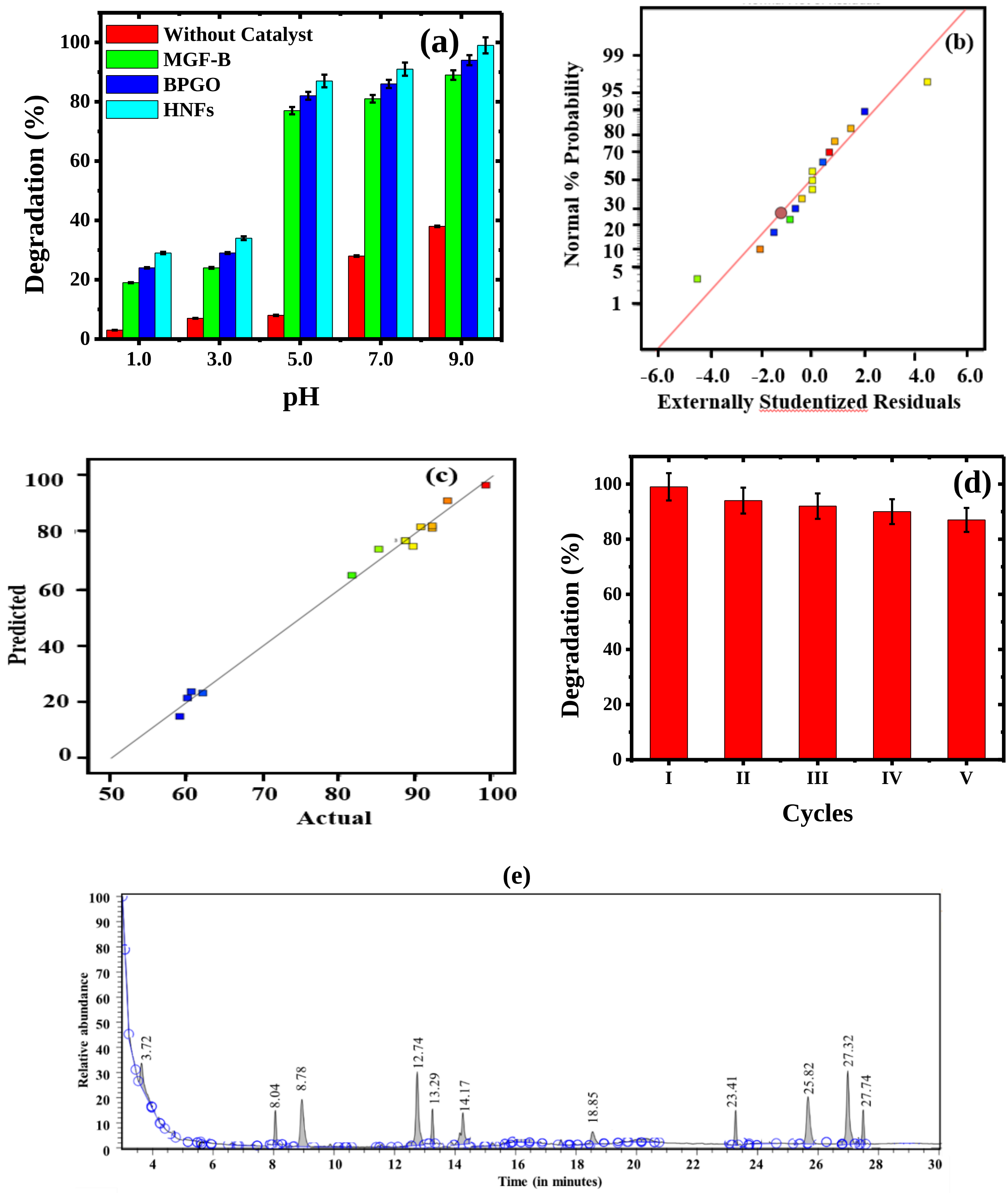


**Figure S6 (a) Photocatalytic degradation of malathion under visible light irradiation (Experimental conditions: HNFs dose-0.2 gL$^{-1}$, irradiation time-2 hours, malathion concentration-2.0 mgL$^{-1}$, Plots of (b) residuals normal probability (c) predicted vs. actual relationship, (d) GC-MS spectra of malathion, (e) Reusability of HNFs for malathion degradation (Experimental conditions: optimum pH, malathion concentration-2.0 mgL$^{-1}$, HNFs dose-0.2 gL$^{-1}$)**

**Table S3: Comparison of Langmuir adsorption capacity**

| S.No. | Adsorbent | Temperature | pH | Adsorbent dose (g $L^{-1}$) | Maximum adsorption capacity (mg$g^{-1}$) | References |
|---|---|---|---|---|---|---|
| **1.** | $MoS_2$ | 298 | 5.0 | 0.8 | 263.6 | [50] |
| **2.** | Fe NPs | 293 | 5.0 | 1.0 | 80.2 | [51] |
| **3.** | Cellulose | 293 | 5.5 | 0.5 | 295.2 | [52] |
| **4.** | Mercapto-modified GO | - | 5.0 | - | 108.0 | [53] |
| **5.** | GO-MgO | 293 | 6.5 | 0.4 | 190.0 | [54] |
| **6.** | Activated carbon | 293 | - | 0.4 | 51.1 | [55] |
| **7.** | Iron oxide- clay-activated carbon composite | 298 | 4.5 | 2.0 | 41.3 | [56] |
| **8.** | Biochar | - | 5.0 | 0.5 | 7.9 | [57] |
| **9.** | GO | 298 | 6.0 | - | 250.0 | [58] |
| **10.** | EDTA-GO | 298 | 6.8 | 0.1 | 479.0 | [59] |
| **11.** | Amino siloxane oligomer-linked GO | 293 | 4-5 | 1.0 | 200.0 | [60] |
| **12.** | HNFs | 293 | 6.0 | 0.4 | 654.23 | In our study |

**Table S4: Thermodynamic parameters**

| | | T ≤ 323 | T > 323 |
|---|---|---|---|
| **$\Delta H^{oa}$ (kJ $mol^{-1}$)** | | 11.39 | -39.41 |
| **$\Delta S^{ob}$ (kJ $mol^{-1}$.K)** | | 0.07 | 0.06 |
| **$\Delta G^{oc}$(kJ $mol^{-1}$)** | **283** | -8.36 | |
| | **288** | -9.04 | |
| | **293** | -10.21 | |
| | **298** | -10.86 | |
| | **303** | -11.14 | |
| | **308** | -11.45 | |
| | **313** | -11.74 | |
| | **318** | -12.02 | |
| | **323** | -12.19 | |
| | **328** | -10.91 | |

[a-] Change in enthalpy, [b-] Change in entropy, [c-] Change in Gibb's free energy

**Table S5: Distribution, selectivity and ionic radii of Pb(II), Cd(II), Zn(II) and Ni(II) ions**

| Heavy metal ion | Using fabricated nanostructure | | Ionic radii (A°) |
|---|---|---|---|
| | $D^a_p$ | $S^b$ | |
| **Pb (II)** | 32.5 | - | 1.19 |
| **Cd (II)** | 9.285 | 3.5 | 0.95 |
| **Zn (II)** | 5.638 | 1.646 | 0.74 |
| **Ni (II)** | 3.928 | 1.435 | 0.71 |

[a]- The value of distribution coefficient (D) is calculated according to the given equation:

$$D = \frac{q}{C_e}$$

[b]- The selective adsorption of Pb(II) ions in the presence of competing metal ions were investigat by the selectivity factor (S) which is determined by:

$$S = \frac{D_p}{D_m}$$

Where q, $C_e$, $D_p$ and $D_m$ are the adsorption capacity (mg/g), equilibrium concentration (mg/ distribution coefficients for Pb (II) and M = Zn (II), Cd (II) and Ni (II) ions.
